# Intrinsic Patterns of Coupling between Correlation and Amplitude of Low-Frequency fMRI Fluctuations Are Disrupted in Degenerative Dementia Mainly due to Functional Disconnection

**DOI:** 10.1371/journal.pone.0120988

**Published:** 2015-04-06

**Authors:** Daniele Mascali, Mauro DiNuzzo, Tommaso Gili, Marta Moraschi, Michela Fratini, Bruno Maraviglia, Laura Serra, Marco Bozzali, Federico Giove

**Affiliations:** 1 Museo Storico della Fisica e Centro Studi e Ricerche Enrico Fermi, Rome, Italy; 2 Dipartimento di Fisica, Sapienza Università di Roma, Rome, Italy; 3 Fondazione Santa Lucia IRCCS, Rome, Italy; 4 Dipartimento di Scienze, Università degli Studi Roma Tre, Rome, Italy; University Of Cambridge, UNITED KINGDOM

## Abstract

Low frequency fluctuations (LFFs) of the BOLD signal are a major discovery in the study of the resting brain with functional magnetic resonance imaging (fMRI). Two fMRI-based measures, functional connectivity (FC), a measure of signal synchronicity, and the amplitude of LFFs (ALFF), a measure of signal periodicity, have been proved to be sensitive to changes induced by several neurological diseases, including degenerative dementia. In spite of the increasing use of these measures, whether and how they are related to each other remains to be elucidated. In this work we used voxel-wise FC and ALFF computed in different frequency bands (slow-5: 0.01-0.027 Hz; slow-4: 0.027-0.073 Hz; and full-band: 0.01-0.073 Hz), in order to assess their relationship in healthy elderly as well as the relevant changes induced by Alzheimer’s Disease (AD) and Mild Cognitive Impairment (MCI). We found that in healthy elderly subjects FC and ALFF are positively correlated in anterior and posterior cingulate cortex (full-band, slow-4 and slow-5), temporal cortex (full-band and slow-5), and in a set of subcortical regions (full-band and slow-4). These correlation patterns between FC and ALFF were absent in either AD or MCI patients. Notably, the loss of correlation between FC and ALFF in the AD group was primarily due to changes in FC rather than in ALFF. Our results indicate that degenerative dementia is characterized by a loss of global connection rather than by a decrease of fluctuation amplitude.

## Introduction

Resting-state functional magnetic resonance imaging (rs-fMRI) has been widely used to investigate the brain function in the absence of a specific task [1]. The resting brain exhibits low frequency (< 0.1 Hz) fluctuations (LFFs) of the blood oxygenation level dependent (BOLD) signal that are synchronized across functionally related and anatomically connected regions. Although the nature of LFFs is not completely understood, resting-state fMRI has improved the characterization of brain functional architecture. Biswal and colleagues were the first to report highly correlated LFFs in the motor cortex in the absence of task [2]. Thereafter, several studies have consistently shown that specific patterns of synchronized LFFs identify different resting-state networks (RSNs), including, but not limited to visual, auditory, attentive and the so-called default-mode network (DMN) [3, 4].

Functional connectivity (FC) characterizes the degree of LFFs synchronization in the resting brain. It has been largely inferred by seed-based standard correlation analysis or alternatively by data-driven approaches, such as independent component analysis (ICA) [5]. These methods are roughly independent from the relative oscillation amplitude, despite its obvious relevance in terms of signal to noise ratio. However, different works have suggested that the amplitude of LFFs *per se* could be of physiological relevance. Biswal and colleagues reported higher values of spectral power in the grey than in the white matter [2]. Recently, Zang and colleagues formalized the resting-state spectral power by introducing a new quantitative measure, namely the amplitude of low-frequency fluctuation (ALFF) [6]. ALFF measures voxel-wisely the total power of a given BOLD time course within the low-frequency band [7, 8].

FC and ALFF have been increasingly used to investigate the brain at rest, although the qualitative and quantitative relationship between them still needs to be fully understood. To the best of our knowledge, one single work examined systematically the relationship between FC and ALFF. Di and colleagues showed a widespread pattern of both positive and negative local correlations between network strength (i.e., ICA-based FC) and ALFF in a healthy elderly population [9]. Moreover, ALFF values from a number of brain regions, such as the medial prefrontal cortex (mPFC), anterior cingulate cortex (ACC), precuneus, basal ganglia, thalamus and insula, were found to be correlated with FC in several independent RSNs. This latter evidence suggests that the coupling between FC and ALFF could be a global feature of the brain, which is relevant because the coupling between FC and ALFF likely depends on the way FC is defined. Indeed, while ALFF is a univariate measure univocally defined for each voxel, FC requires the definition of a relation (e.g. Pearson correlation) between features of different voxels or areas. Therefore, a global, voxel-wise measure of FC is perhaps the most appropriate to best match the nature of ALFF and to further investigate the properties of FC vs ALFF coupling.

In principle, temporal correlation should be independent from the amplitude of compared signals. However, the fact that several pathological states are characterized by partially co-localized changes in FC and ALFF suggests the existence of a specific relationship between them. For example, Alzheimer's disease (AD) and mild cognitive impairment (MCI; a condition widely regarded as a prodromal stage of AD) patients show altered patterns of FC in specific DMN areas, i.e. posterior cingulate cortex (PCC)/precuneus, lateral temporal, parietal and medial frontal cortex, as well as hippocampus [10–16]. Similarly, widespread alterations of ALFF have been reported in AD [12, 17, 18] and MCI [18–20] patients. Although a growing body of evidence exists to support impaired LFFs in dementias, there is no systematic study exploring the concomitant changes of FC and ALFF as an effect of neurodegeneration. Indeed, the observed FC reduction in dementia could be related to (i) a concomitant reduction of ALFF towards the noise level or (ii) a loss of synchronization between areas showing unaltered, or possibly increased, fluctuation amplitude.

Although the majority of resting state studies have focused on a relative wide range of frequency (e.g., 0.01–0.1Hz), converging evidence suggests that a finer band subdivision might provide some additional information. Zuo and colleagues, extending the Buzaski framework on neuronal oscillator classes [21] to BOLD LFFs, divided the BOLD power spectrum into four distinct frequency ranges, namely slow-5 (0.01–0.027 Hz), slow-4 (0.027–0.073 Hz), slow-3 (0.073–0.198) and slow-2 (0.198–0.25 Hz) [8]. While slow-3 and slow-2 mainly reflect cardiac- and respiratory-related effects, slow-5 and slow-4 appear as stable parameters across scans [8] with preferential spatial patterns of oscillation [7, 8]. Moreover, frequency-dependent changes in ALFF have been reported in MCI [22, 23] and in AD [24] as well as in other neurological or psychiatric disorders, such as Parkinson’s disease [25] and schizophrenia [26]. Frequency-specific patterns of BOLD fluctuations were also found to be associated with specific personality traits [27]. In addition to ALFF, FC has also been characterized using a finer band analysis. Indeed, the topological properties of functional brain networks have shown frequency-dependent features, with slow-4 band displaying higher small-world metrics and test-retest reliability as compared to slow-5 [28]. Band-subdivision has also significantly improved the MCI classification accuracy using graph-theory-based FC [29]. The origin and the specific physiological function of each frequency band is presently still unknown. Yet, from the spectral properties of FC and ALFF taken separately, we hypothesize that the relationship between ALFF and FC could be frequency-dependent.

In the present work, we sought to investigate the relationship between ALFF and global FC with a voxel-based approach in the healthy and diseased brain. The main aim of the study was to assess whether the coupling between ALFF and FC depends on anatomical brain localization, frequency band, and pathological conditions. For this purpose, we performed rs-fMRI employing degenerative dementia as a pathological model, and enrolling patients with both AD and MCI. Assuming the existence of a connection between ALFF and the average FC in each voxel with the whole brain, we used a generalized linear model (GLM) to systematically assess to what extent the voxel-wise variance of FC across subjects is explained by the amplitude of the underlying oscillations.

## Methods

### Subjects

Ten patients with a diagnosis of probable AD by NINCDS-ADRDA consensus criteria [30], 10 amnestic MCI [31] patients and 10 healthy elderly subjects (HC) were recruited for this study. A general cognitive evaluation was obtained using the Mini-Mental State Examination (MMSE).

Age, education and MMSE score distributions were compared among groups via one-way analysis of variance (ANOVA), while a chi-square test was applied to compare gender distribution. Where indicated, two-sample, two-tailed t-tests were performed as post-hoc analyses.

The current study was approved by the ethics committee of Santa Lucia Foundation. Every recruited subject (or his/her responsible guardians if incapable) gave written consent before MR study initiation.

### Data acquisition

Data were acquired on a 3T MRI system (Magnetom Allegra, Siemens, Erlangen, Germany). All subjects underwent a resting state fMRI scan using a echo planar imaging (EPI) sequence with the following parameters: TR = 2080 ms, TE = 30 ms, 32 axial slices parallel to AC-PC plane, matrix = 64 x 64, in plane resolution = 3x3 mm^2^, slice thickness = 2.5 mm, 50% skip, flip angle = 70°. Resting scans lasted for 7 minutes and 20 seconds for a total of 220 volumes during which subjects were instructed to keep their eyes closed, to not think of anything in particular and to refrain from falling asleep. A T1-weighted three-dimensional modified driven equilibrium Fourier transform scan (MDEFT, [32]) was acquired for each subject for anatomical localization purposes and for grey matter (GM) volumetry; the parameters were as follows: TR = 1338 ms, TE = 2.4 ms, TI = 910 ms, flip angle = 15°,matrix = 256 x 224 x 176, FOV = 256 x 224 mm^2^, slice thickness = 1 mm, total scan time = 12 min. Fluid attenuated inversion recovery (FLAIR) images (TR = 8170 ms, TE = 96 ms, TI = 2100 ms) were also acquired from all subjects to exclude the presence of remarkable signs suggestive for cerebro-vascular disease, as previously described [33].

### Data preprocessing

Functional images were preprocessed using Connectivity toolbox (CONN: functional connectivity toolbox [34]). The first four volumes were discarded to allow signal and scanner stabilization. Images were slice-time corrected and realigned to the first image. For each subject, the mean EPI image, obtained from the realignment step, was used as source image to estimate the transformation parameters to match the functional images with the high resolution T1 volume. Then, all coregistered volumes were normalized into Montreal Neurological Institute (MNI) space coordinates (voxel size: 2x2x2 mm^3^). Normalized images were then smoothed using an 8x8x8 mm^3^ full width at half maximum (FWHM) Gaussian kernel.

Before statistical analyses, data from each subject underwent physiological noise mitigation. The six parameters of realignment and the first five eigenvectors of the PCA decomposition of the EPI time course averaged over cerebrospinal fluid (CSF) and white matter (WM) were regressed out, following aCompCor approach for physiological noise removal [35]. Data were then detrended and filtered in three different frequency ranges: 1) full-band: 0.01–0.073 Hz. 2) Slow-5: 0.01–0.027 Hz. 3) Slow-4: 0.027–0.073 Hz [8].

### Grey matter volumetry

The T1-weighted MDEFT images were processed using the VBM protocol [36] implemented in SPM8 (http://www.fil.ion.ucl.ac.uk/spm/), which consists of an iterative combination of segmentations and normalizations to produce a GM probability map [36] in MNI standard space for every subject. In order to compensate for compression or expansion which might occur during warping of images to match the template, GM maps were “modulated” by multiplying the intensity of each voxel in the final images by the Jacobian determinant of the transformation, corresponding to its relative volume before and after warping [37]. All data were then smoothed using a 12x12x12 mm^3^ FWHM Gaussian kernel, and finally the GM volume (GMV) was computed by summing the relevant modulated partition, multiplied by the voxel volume.

### Functional connectivity

Voxel-wise connectivity analysis was carried out using 3dTcorrMap (AFNI package, [38]) as previously described by others [39]. For each voxel, Pearson correlation coefficients between each voxel and all other voxels of the brain were computed; then, after a z-Fisher transformation, these coefficients were averaged. The mean value thus obtained expresses the strength of connectivity between each voxel and the rest of the brain (i.e., a measure of global connectivity). Map of global connectivity were produced for each of the three frequency ranges.

Voxel-wise based FC computation is less sensitive than FC obtained by using a seed-based approach, which has been adopted among the others by Di and colleagues [9]. Nonetheless, the former computation has a higher intrinsic spatial resolution, and it is independent from the arbitrary choice of regions of interest (ROIs). Thus, we adopted a voxel-wise computation of FC as it is closer to the nature of ALFF, being inherently able to map the average value of FC in each single voxel.

### ALFF

For each voxel in the brain the filtered time series (0.01–0.073 Hz) was transformed into the frequency domain using a Fast Fourier Transform (FFT) algorithm (3dPeriodogram; AFNI package, [38]). The obtained power spectral density was square rooted and averaged in the three frequency bands of interest: full-band, Slow-5 and Slow-4. ALFF maps of each subject were transformed into z-scores [8].

### Statistical analysis

For each subject group, the mean effects of ALFF and FC were assessed via one-sample, two-tailed t-tests (p < 0.05 corrected for multiple comparisons; see below). Distribution of abnormal ALFF and FC values across groups were evaluated via one-way ANOVA (p < 0.05, corr.).

Z-transformed correlation and ALFF maps from each subject were entered in a second level analysis to assess whether the amplitude of oscillations affects the correlation strength. GLM was applied voxel-wise, considering the FC and ALFF z-scores as dependent and independent variables respectively. Age, gender, education and GM volume were standardized and entered in the model as nuisance covariates. The statistical significance of the regression FC vs ALFF was assessed in each group by one sample, two-tailed t-tests. Within the areas where the regression returned significant coupling in HC, group differences in the strength of correlation were assessed between all experimental groups (i.e., HC, AD and MCI patients) by two-sample, two-tailed t-tests. Model estimation and t-contrasts were replicated for each frequency range. The calculation was carried out with custom software implemented in Matlab R2012a (The Mathworks Inc, Natick, Massachusetts, USA).

Statistical threshold was set to p < 0.05 after correction for multiple comparison, performed by Monte Carlo simulations (AlphaSim; AFNI package, [38]). The corrected threshold of p < 0.05 corresponds to a single voxel threshold of p < 0.005 with a minimum cluster size of 157 voxels for whole-brain inference. For mask based inferences the minimum cluster size was set around 40 voxels, depending on the mask size.

## Results

Principal demographic, clinical data and GMV estimation are reported in Table 1. Patients and controls were matched for age (ANOVA, f = 1.5, p > 0.2) and gender (chi-square test: x^2^ = 1.9, p > 0.3), although patients were less educated than controls (t-tests: AD vs HC, t = -4.0, p < 0.001; MCI vs HC, t = -2.3, p < 0.05). As expected, MMSE scores were significantly different between all groups (t-tests: AD vs HC, t = -6.5, p < 0.001; MCI vs HC, t = -4.6, p < 0.001; MCI vs AD: t = 3.1 p < 0.05). AD patients showed a significant reduction of GMV when compared to both HC and MCI patients (t-tests: AD vs HC, t = -3.9, p < 0.001; AD vs MCI, t = -3.6, p < 0.05). Conversely, despite the presence of a reduction trend in GMV, volumes were not significantly different between MCI patients and HC (t-test: MCI vs HC, t = -1.4, p > 0.1).

**Table 1:** Principal characteristics of studied subjects.

	AD	MCI	HC	P-value
**N**	10	10	10	
**Gender (M/F)**	4/6	6/4	7/3	0.387^a^
**Age (years)**	72.3 ± 8.3	70.7 ± 7.1	66.0 ± 9.6	0.235^b^
**Education (years)**	8.6 ± 3.6**	11.1 ± 3.5*	14.5 ± 3.0	0.002^b^
**MMSE score**	21.5 ± 3.7** ^†^	25.8 ± 2.3**	29.30 ± 0.67	<0.001^b^
**Grey matter volume (dl)**	5.20 ± 0.51** ^†^	6.10 ± 0.61	6.6 ± 1.0	<0.001^b^

Data presented as mean ± SD. AD and MCI data were tested against HC data (two-sample two-tailed t-test; * p < 0.05; ** p < 0.001); as well as against each other (^†^ p < 0.05). AD, Alzheimer’s disease; MCI, mild cognitive impairment; HC, healthy control; MMSE, Mini mental State Examination.

^a^ The p-value was obtained by chi-square test.

^b^ The p-value was obtained by one-way ANOVA test.

**Fig 1:**
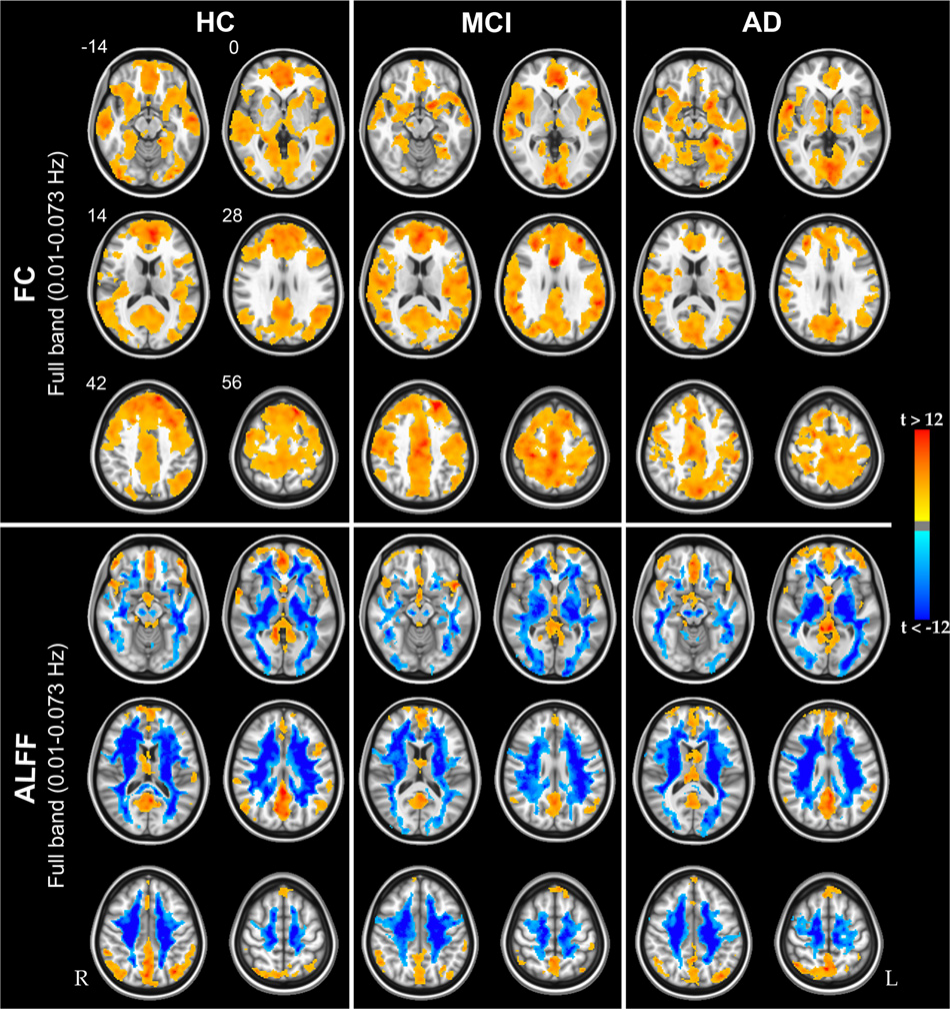
Functional connectivity and amplitude of low frequency fluctuations group results. Group-level *t*-statistic maps showing significantly detectable FC (upper panel) and ALFF (lower panel) in the full-band frequency range. The three groups of subjects, HC, MCI and AD patients, are reported from left to right. Hot and cold colors encode for positive and negative group effect, respectively. Results were obtained via one-sample, two-tailed, t-tests (|t| > 3.7; p<0.05, corrected). Of note, despite it is expected to find negative ALFF values in white matter [8], this effect was striking in our results and it is primarily caused by the CompCor approach for noise mitigation [35]. The numbers next to the images refers to z coordinates in the MNI space. R, right; L, left.

FC and ALFF maps in the full-band range are shown for each studied group in Fig 1. Voxel-wise FC analysis reproduced patterns of connectivity in agreement with those reported by others using similar measures of global FC [39, 40]. In particular, our results showed strongly connected regions belonging to the DMN, such as the precuneus/PCC, mPFC/ ventral ACC, and the inferior temporal/parietal cortex. These patterns are more overtly appreciated using a higher statistical threshold (S1 Fig). No negative FC was found in any of the studied groups. Similarly to FC, high ALFF values were found in the GM, primarily in the precuneus/PCC and in the mPFC.

**Fig 2:**
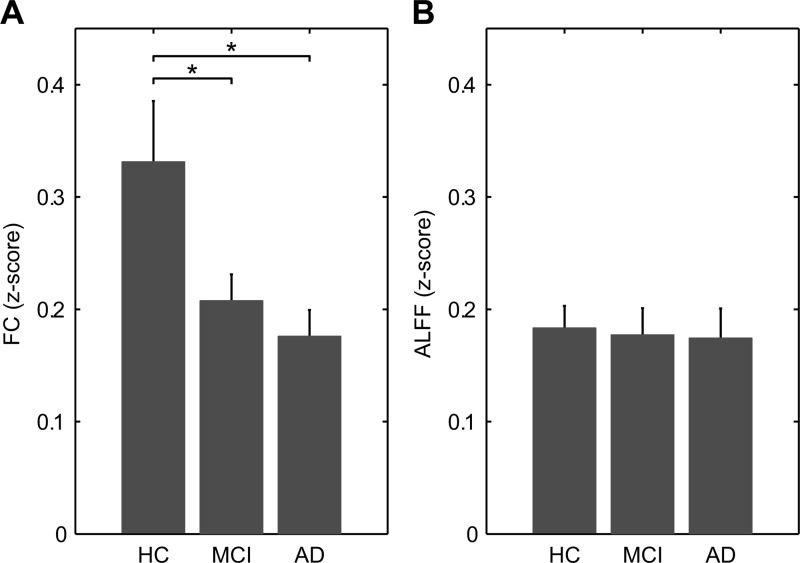
Average grey matter functional connectivity and amplitude of low frequency fluctuations. Both measures were extracted from a common grey matter mask. This mask was obtained averaging grey matter probability maps (from the segmentation step) across all subjects and thresholding the resulting map at 0.75. (A) Group average FC z-score. Diseased groups show significant reduction compared to HC. (B) Group average ALFF z-score. No significant difference among groups were found. In particular, MCI and AD are indistinguishable from healthy subjects (p > 0.8 and p > 0.7, respectively). * p < 0.05 (two-sample, two-tailed *t*-test).

Group averages of FC z-score in the GM (Fig 2A) showed a significantly reduced connectivity in both AD and MCI patients compared to HC (two-sample, two-tailed t-tests: HC vs AD, t = 2.8, p < 0.05; HC vs MCI, t = 2.2, p < 0.05; MCI vs AD, t = 1.0, p > 0.3). On the contrary, group averages of ALFF z-scores in the GM mask (Fig 2B) revealed no significant differences among groups (one-way ANOVA test: f = 0.04, p > 0.9). Voxel-wise one-way ANOVA and relative post-hoc analyses of FC and ALFF corroborated these whole-brain trends among groups (S2 Fig).

**Fig 3:**
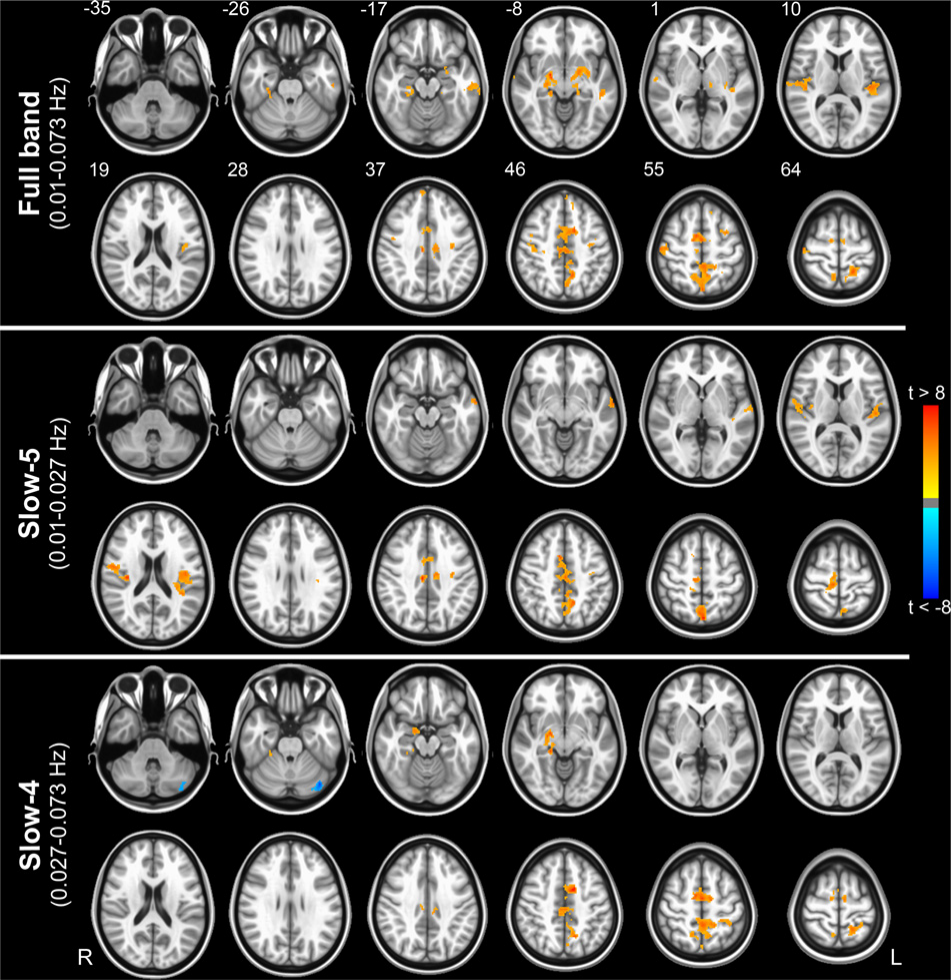
Voxel-wise correlation between functional connectivity and amplitude of low frequency fluctuations in the healthy brain. Color-coded t-statistic maps showing significantly detectable correlation between FC and ALFF for the full-band (upper panel), slow-5 (middle panel) and slow-4 (bottom panel) frequency range, considering only the HC group. Hot and cold colors encode for positive and negative correlations, respectively. Results were obtained via a t-contrast (HC > 0) in the “FC vs ALFF” model (|t| > 3.2; p < 0.05, corrected). The numbers next to the images refer to z coordinates in the MNI space. R, right; L, left.

Having confirmed previous results on the overall spatial distribution of FC and ALFF in healthy controls and patients, we next examined the results of the linear regression between FC and ALFF within each group. Brain regions in which the correlation strength was significantly explained by the amplitude of oscillations in HC are shown in Fig 3 for each considered frequency band. For the full-band range, these areas included the cingulate cortex and precuneus, the superior temporal cortex/insula, the medial frontal cortex, the thalamus, the lentiform nucleus and the parahippocampal cortex (Fig 3, top). Common patterns for the slow-5 and slow-4 bands (Fig 3, middle and bottom, respectively) were found in the cingulate cortex and precuneus. The slow-5 band showed also additional FC vs ALFF correlation patterns in the superior temporal cortex/insula, while the slow-4 band showed some lateralized (right) subcortical effect (thalamus, lentiform nucleus). All the regions reported above showed a positive relationship between FC and ALFF. One region only, located in left cerebellum, showed a negative association between FC and ALFF, which was isolated to the slow-4 band. However, since the cerebellum barely fell in the bottom edge of EPI field, the area showed a lower signal to noise ratio and suboptimal normalization when compared to the cerebrum. Therefore, this negative correlation is likely due to artifacts. Remarkably, in contrast to HC, neither AD nor MCI did show any area with significant regression between FC and ALFF (p > 0.05 cor.).

**Fig 4:**
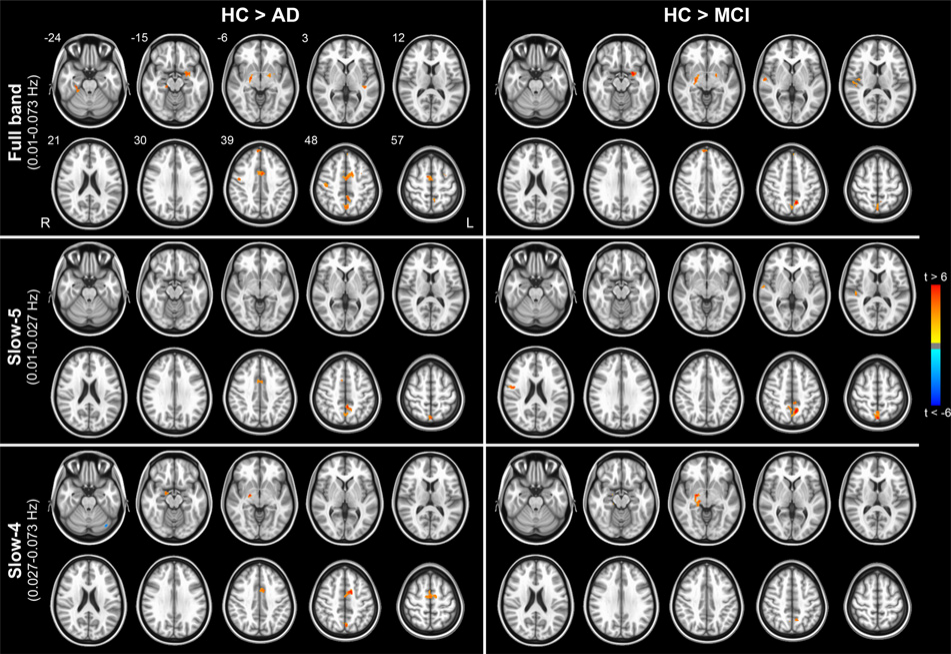
Pathology-induced changes in the coupling between functional connectivity and the amplitude of low frequency fluctuations. Color-coded t-statistic maps showing significantly detectable changes in the correlation between FC and ALFF comparing HC to AD patients (left) and MCI patients (right). Comparing AD to MCI lead no significant cluster. Tests were separately carried out for the full-band (upper panel), slow-5 (middle panel) and slow-4 (bottom panel) frequency range. Only the brain areas in which was found a statistically significant relationship in HC were considered in these tests (i.e., t-maps masked with patterns in Fig 3). Results were obtained via t-contrasts (HC > AD and HC > MCI) in the “FC vs ALFF” model (|t| > 2.8; p < 0.05, corrected). Hot and cold colors encode for HC > AD/MCI and HC < AD/MCI, respectively. For the details of the involved regions, see Tables 2 and 3. The numbers next to the images refer to z coordinates in the MNI space. R, right; L, left.

We then investigated the specific effect of disease in those areas showing significant FC vs ALFF correlation in healthy subjects (i.e., between-group comparisons in areas shown in Fig 3). This analysis demonstrated the local effect of disease on the strength of the FC vs ALFF coupling (Fig 4 left, AD patients; Fig 4 right, MCI patients). Specific cluster locations resulting from t-test HC vs AD and HC vs MCI are reported in Tables 2 and 3, respectively. No significant patterns resulted from the comparison of AD vs MCI (p > 0.05 cor.).

**Table 2:** Regional differences in the FC vs ALFF coupling comparing HC to AD.

Frequency range	Brain Region	Hemisphere	Vol (voxels)	MNI coordinates			Peak t-value
				x	y	z	
**Full-band**	Cingulate gyrus / Supplementary motor area	B	360	0	-6	52	5.97
	Precuneus	B	171	-2	-46	50	5.38
	Lentiform Nucleus/ Parahippocampal gyrus	L	113	-20	0	-10	5.26
	Parahippocampal gyrus	R	106	30	-28	-20	5.71
	Precentral Gyrus	R	96	46	-8	42	5.66
	Lentiform Nucleus/Lateral Globus Pallidus / Amygdala	R	73	20	-6	-8	6.80
	Middle Frontal Gyrus	L	66	-32	6	54	6.79
	Medial Frontal Gyrus	B	50	4	54	42	5.33
	Superior Temporal Gyrus	L	47	-40	-28	2	4.79
**Slow-5**	Precuneus	B	93	-4	-46	48	5.73
	Precuneus	B	92	0	-70	58	4.85
	Precentral Gyrus	R	86	16	-38	70	4.83
	Cingulate gyrus	B	75	8	10	46	6.31
**Slow-4**	Cingulate gyrus / Supplementary motor area	B	424	-10	4	46	8.27
	Globus pallidus / Amygdala	R	92	22	-10	-6	7.96
	Cerebellum	L	85	-42	-72	-30	-5.89
	Precuneus	B	85	-2	-70	48	5.04

Regions showing significant pathology-induced (Alzheimer’s disease) changes in the coupling between functional connectivity and the amplitude of low-frequency fluctuations (t-test: HC > AD). B, bilateral; L, left; R, right.

**Table 3:** Regional differences in the FC vs ALFF coupling comparing HC to MCI.

Frequency range	Brain Region	Hemisphere	Vol (voxels)	MNI coordinates			Peak t-value
				x	y	z	
**Full-band**	Precuneus	B	278	-8	-54	50	7.37
	Superior Temporal Gyrus / Insula	R	139	44	-26	8	5.77
	Parahippocampal gyrus / Amygdala	L	99	-22	2	-12	7.49
	Lentiform Nucleus / Amygdala	R	99	20	-8	-10	5.77
	Medial Frontal Gyrus	R	46	4	54	42	5.70
**Slow-5**	Precuneus	B	375	-8	-56	48	6.89
	Insula / Precentral Gyrus	R	66	46	-2	18	5.89
	Superior Temporal Gyrus	R	54	64	-12	6	4.89
	Cingulate gyrus	L	45	-4	-40	42	4.97
	Postcentral Gyrus / Insula	R	41	50	-22	18	4.74
**Slow-4**	Globus Pallidus / Amygdala / Hippocampus	R	164	22	-10	-6	7.99
	Cerebellum (Declive)	L	69	-42	-72	-30	-6.29
	Precuneus	L	65	-12	-58	46	5.87
	Precuneus	R	62	6	-66	50	4.76

Regions showing significant pathology-induced (mild cognitive impairment) changes in the coupling between functional connectivity and the amplitude of low-frequency fluctuations (t-test: HC > MCI). B, bilateral; L, left; R, right.

**Fig 5:**
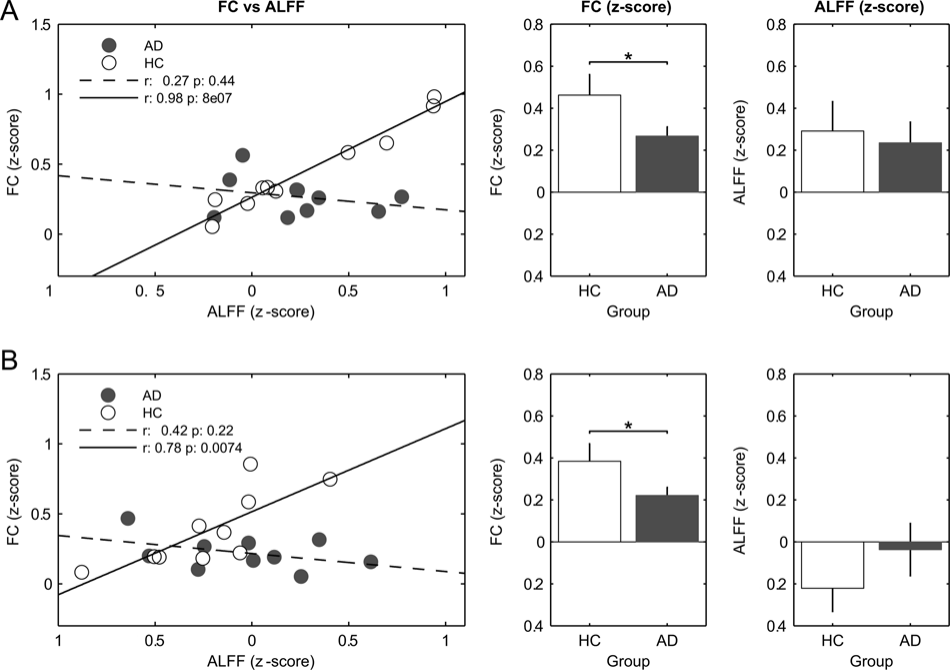
Trend of functional connectivity vs amplitude of low frequency fluctuations along with respective averaged values. FC and ALFF values were averaged in the significant clusters resulting from t-contrast HC vs AD in the full-band analysis (i.e., Fig 4, top row, left column). The significant clusters were grouped in cortical (A) and subcortical (B) regions. For the details of the regions, see Table 2. * p < 0.05 (two-sample, one-tailed t-test).

Finally, to assess the origin of the reduced correlation between FC and ALFF, their values were averaged over each of the ROIs reported in Tables 2 and 3. We found several ROIs showing a significant reduction of FC in patients as compared to HC; on the contrary, no ROIs showed either a significant reduction in ALFF or any other clear trend (S3 Fig). Finally, according to their location the voxels included in all the ROIs were classified as cortical or subcortical, and ALFF and FC values were averaged subject by subject in these two macro-areas. In both macro-areas, and for the full-band frequency range, there was a disruption of the correlation between FC and ALFF in AD compared to HC (Fig 5, left plots). In both, cortical (Fig 5A) and subcortical (Fig 5B) regions, a significant reduction in FC, but not in ALFF, was proportional to the drop in correlation between these two measures. All other tests (i.e., in other frequency bands and including the MCI group) showed a similar trend towards reduction of FC, although they did not reach the full statistical significance.

## Discussion

### FC vs ALFF in healthy population

The present study revealed specific spatial and frequency-dependent patterns of positive regression coefficients between global FC and ALFF in healthy subjects (Fig 3). Involved regions can be assembled in three main groups, namely temporal, parietal and subcortical areas. Results demonstrated that these brain areas are characterized by voxels whose levels of oscillation amplitude are directly associated to the strength of their global connection. Interestingly, many of these areas are known to belong to the most globally connected regions of the brain. For example, the cingulate cortex belongs to the DMN, which is characterized by a high degree of functional [39] and anatomical [41] connections. Similarly, the insula, thalamus and basal ganglia are implicated in several long range connections [39, 42, 43]. These results suggest therefore that highly connected regions in the brain are also regions whose connectivity is more sensitive to variations of the underlying fluctuation amplitude.

Our results in the full frequency band are broadly consistent with those obtained by Di and colleagues, even though we did not find any negative correlation pattern (with the exception of the cerebellum, as discussed in the results section) [9]. However, while Di and colleagues adopted the ICA and seed-based analysis to compute FC, we privileged a voxel-wise global measure of connectivity which is independent from a specific network decided a priori. This means that FC can be directly and unequivocally compared with ALFF in each voxel. Accordingly, our results demonstrate that FC vs ALFF coupling is network independent and may be regarded as global characteristic of each single voxel.

The separation of LFFs in two distinct frequency ranges (slow-5 and slow-4) demonstrated that the relationship between ALFF and FC is sensitive to the frequency range, and specific patterns of correlation could be identified for slow-5 and slow-4 bands. Although the full-band analysis returned essentially the same areas highlighted by the sub-band analysis, the slow-5 band was clearly more sensitive for couplings in temporal lobe regions. Moreover, slow-5 primarily identified cortical regions, while slow-4 included both cortical and subcortical regions. These results are in agreement with recent studies, which reported that the fluctuations at the higher bound of LFFs are mainly localized in subcortical areas [7, 8]. The different patterns are likely due to the different oscillatory mechanisms underlying these two frequency bands. Previous works suggested that the limited speed of signal propagation, primarily due to synaptic delay and axonal conduction, along with physical constraints (i.e., the size) of the engaged neuronal network may account for the different periods of oscillation. Under this assumption, large neuronal assemblies may result in a larger period of electrophysiological oscillation if compared to small neuronal space [21, 44]. Thus, according to this view the relative large size of cortical as compared to subcortical structures might explain the different patterns observed at different bands. Alternatively, the different coupling in slow-4 and slow-5 bands could arise from different synaptic, functional or cytoarchitectonic features of the different areas [7, 45]. Nonetheless, it still remains largely unclear how the BOLD signal depends on the spontaneous neuronal activity observed at different spatiotemporal scales. Moreover, the frequency window of BOLD signal for the slow-5 and slow-4 components is seemingly too narrow for discriminating between different frequency/size features of neuronal assemblies.

A higher fluctuation amplitude in slow-4 as compared to slow-5 has been observed with a symmetrical pattern in the basal ganglia [8]. In contrast, we found here a lateralization of the FC vs ALFF coupling in slow-4 band of subcortical areas. Since the coupling in those regions was symmetrically preserved in the full-band analysis, slow-4 lateralization pattern might be due to a suboptimal spectral division [45]. Indeed, the use of poorer information (nearly half of the spectrum) as well as the higher amount of noise which is present at lower frequencies, may result in a reduced statistical power.

Both the spatial specificity and the frequency dependence of the coupling between FC and ALFF suggest that the latter measure plays an active role in the generation of synchronization patterns and it is not a simple prerequisite for the emergence of FC. Indeed, the amplitude of BOLD low frequency fluctuations is of physiological relevance, as indicated by many works reporting a coupling between electrophysiological signals and spontaneous BOLD LFFs [46–49]. ALFF was also found to be linearly related to the metabolic consumption of glucose [50]. Moreover, the reduction of low-frequency EEG power from childhood to adulthood, a well-known feature of brain maturation, is accompanied by concurrent reductions in spontaneous BOLD power [51]. Spontaneous BOLD fluctuations were also reported to account for inter-trial variability in behavior [52] and to be more capable than FC in discriminating between different resting conditions (e.g., eyes-open vs eyes-closed) [53]. This evidence suggests that the magnitude of fluctuations provides information on the neuronal workload of the underlying brain area. In this context, the FC vs ALFF correlation pattern we reported here is likely to have a physiological meaning, by expressing local neuronal activity and local strength of global connectivity together.

On the other hand, a non-neuronal origin of the FC vs ALFF pattern cannot be excluded a priori. In principle, a common source of physiological noise could also account for their positive correlation pattern. Cardiac and respiratory fluctuations, spontaneous oscillation in carbon dioxide, and many other physiological phenomena have been identified or proposed as source of BOLD fluctuations [54–59]. For example, ALFF has been shown to correlate with BOLD response in breath holding (BH) task, suggesting that the spectral amplitude of LFFs might be used to scale task-related BOLD signal to account for variability in vascular reactivity [60, 61]. However, previously reported regions of high correlation between ALFF and BH-BOLD response (e.g., cerebellum, midbrain, inferior occipital gyrus, PCC and precuneus) are only minimally overlapped to the correlation pattern that we observed in the current work. This suggests phenomena like vascular reactivity may have only marginally contributed to our results [61].

### FC vs ALFF in degenerative dementia

The comparison between patients and healthy controls revealed a widespread reduction of the FC vs ALFF coupling, at all frequency bands and in both patient groups, AD and MCI (Fig 4). Additionally, such a disruption of correlation patterns was primarily due to a reduction in FC, i.e. loss of fluctuation synchronization rather than to a change in ALFF, in both cortical and subcortical areas (Fig 5).

Different works have reported decreased FC in AD and MCI patients with both seed-based and data driven-analyses [62], especially in the hippocampus, PCC and ACC [16]. These findings are consistent with the reduction trend across groups we reported here in the average GM FC (Fig 2A), as well as shown in voxel-level analysis (S2 Fig). Conversely, we did not observe any significant trend of ALFF values either in the GM (Fig 2B) or in the voxel-level analysis (S2 Fig), except for a small lateralized frontal region with reduced ALFF in both AD and MCI patients. Several works have reported a heterogeneous set of brain regions with abnormal, either increased or decreased, ALFF values in dementia [17, 18, 20, 22], which may reflect the progression of the pathology [63]. Altogether, our and other results support the existence of a complex relationship between AD pathology, neuronal excitability and the underlying vascular and metabolic variables, possibly in a region-dependent manner ([64], and references therein).

We observed that the normal pattern of correlations between FC and ALFF (Fig 3) is disrupted in patients with MCI and AD. Strikingly, in those regions where the correlation FC vs ALFF is altered in patients compared to controls, this phenomenon is related to a loss of synchrony of the oscillations in the presence of preserved oscillation amplitude (Fig 5). This finding indicates that changes of ALFF and FC induced by pathology are partially disentangled, possibly reflecting distinct pathophysiological phenomena. Moreover, our results suggest that the dementia-related changes primarily affect the synchronization of LFFs rather than the oscillation amplitude.

The anatomical pattern of significant FC vs ALFF coupling observed in HC was found to be disrupted as an effect of AD pathology, as shown by group comparisons. Moreover, when considering the overall FC vs ALFF uncoupling, such a disruption was already detectable at the stage of MCI, indicating such a parameter to be sensitive to early pathophysiological aspects of the disease. Further studies on larger populations are needed to clarify the potential diagnostic value of this biomarker. It is possible that the involved regions could have a key role for the maintenance of the ongoing activity (as it is suggested by their partially overlap with the DMN). In the pathological brain, these key regions could be forced to maintain a normal level of activity that, due to the disease, does not produce a comparable degree of synchronization with other brain regions.

## Conclusions

The present resting-state fMRI study revealed that ALFF is linearly related to FC in spatially segregated patterns that are consistent in a healthy elderly population. This relationship is dependent on the specific range of frequencies within the low-frequency band explored in FC studies. The relationship between FC and ALFF is disrupted in AD as well as MCI, in all the studied frequency bands. According to our findings, this is likely due to a loss of fluctuation synchrony among the involved brain areas rather than to altered local activity.

In conclusion, this study suggests that the correlation patterns between ALFF and FC have a neurophysiological correlate, but that ALFF and FC convey partially distinct information, especially as far as the development of changes induced by pathology is involved.

## Supporting Information

S1 Fig Functional connectivity of HC at high threshold level. Group-level t-statistic map showing significant FC on HC in the full-band frequency range. Map result is the same reported in Fig 1, but here is showed at higher threshold level (one sample t-test: t > 6.59, p < 5*10^-5^) for easier identification of most globally connected regions.
(TIF)

S2 Fig Pathology-induced changes in functional connectivity and in amplitude of low frequency fluctuations. (A) f-statistic maps showing significant detectable changes of FC (hot colors) and ALFF values (cold colors) among the three groups of subjects (HC, MCI and AD). Of note, only one lateralized frontal region showed a significant change in ALFF among groups. Results were obtained via one-way ANOVA, separately accomplished for FC and ALFF data. The statistical threshold was set at f > 6.48 (p < 0.05, corrected). (B-E) Post-hoc analyses of the most relevant clusters belonging to FC (B-D) and ALFF (E) changes. For each cluster, group comparisons were performed via two-sample, two-tailed t-tests on the voxel showing the local maximum of f-values. *, p < 0.05; **, p < 0.01; ***, p < 0.001; A, anterior; P, posterior.
(TIF)

S3 Fig FC and ALFF mean values in brain regions showing pathology-induced FC-ALFF uncoupling. FC and ALFF values were averaged in the significant clusters resulting from t-contrasts HC vs AD (A) and HC vs MCI (B) both computed in the FC vs ALFF full-band model (see Fig 4). For the details of the regions, see Tables 2 and 3. *, p < 0.05; **, p < 0.01 (two-sample, one-tailed t-test); Cg, cingulate gyrus; SMA, supplementary motor area; PCu, precuneus; LN, lentiform nucleus; PHg, parahippocampal gyrus; PrG, precentral gyrus; LGP, lateral globus pallidus; Am, amygdala; MiFg, middle frontal gyrus; MeFg, medial frontal gyrus; STg, superior temporal gyrus; In, Insula.
(TIF)
