# Supplementary figures and images for: Intrinsic Patterns of Coupling between Correlation and Amplitude of Low-Frequency fMRI Fluctuations Are Disrupted in Degenerative Dementia Mainly due to Functional Disconnection
Source: PLoS One. 2015 Apr 6;10(4):e0120988. doi: 10.1371/journal.pone.0120988 (PMC4386762; doi:10.1371/journal.pone.0120988)

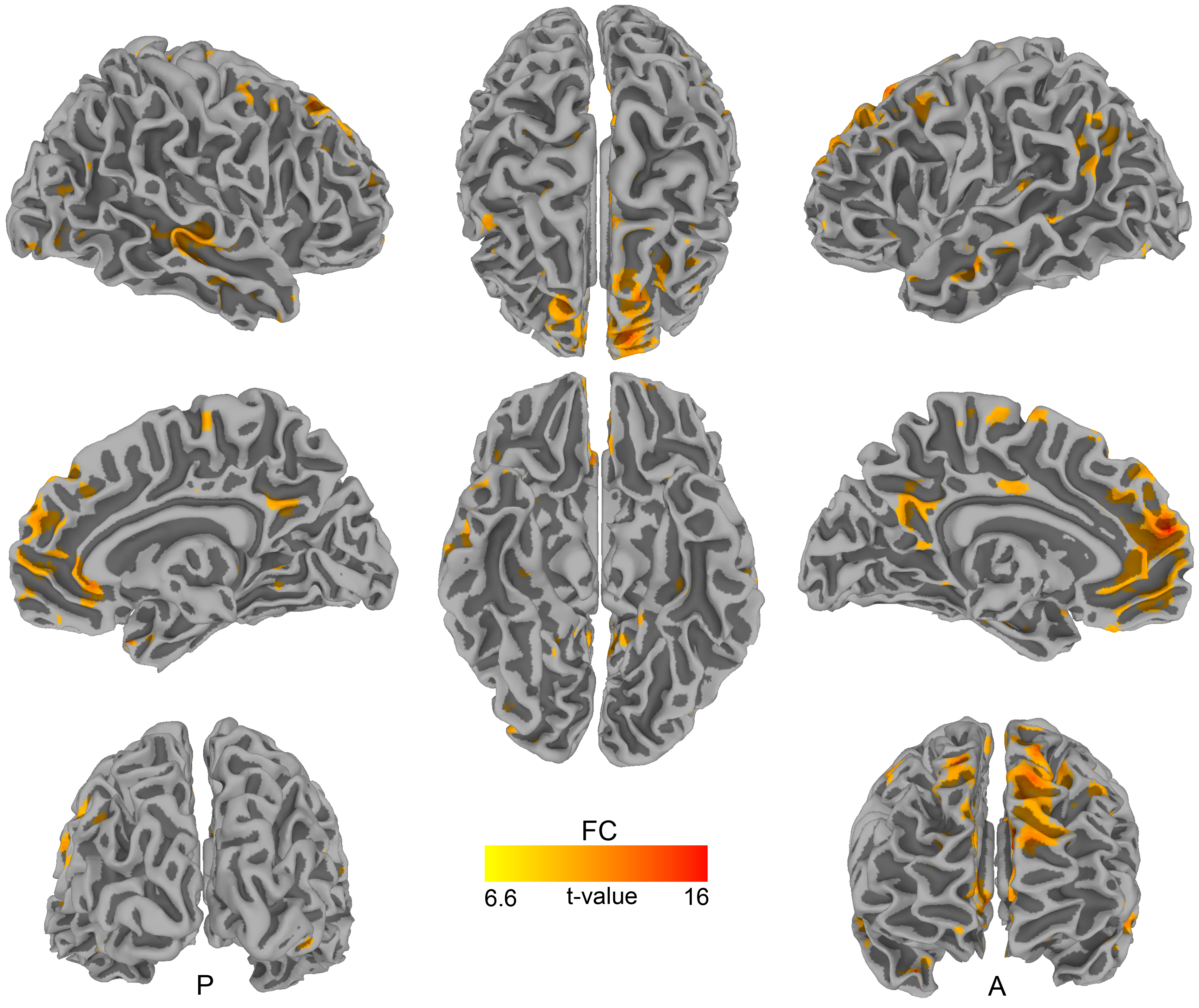

Supplement: S1 Fig — Group-level t-statistic map showing significant FC on HC in the full-band frequency range. Map result is the same reported in Fig 1, but here is showed at higher threshold level (one sample t-test: t > 6.59, p < 5*10-5) for easier identification of most globally connected regions. (TIF) [file pone.0120988.s001.tif]

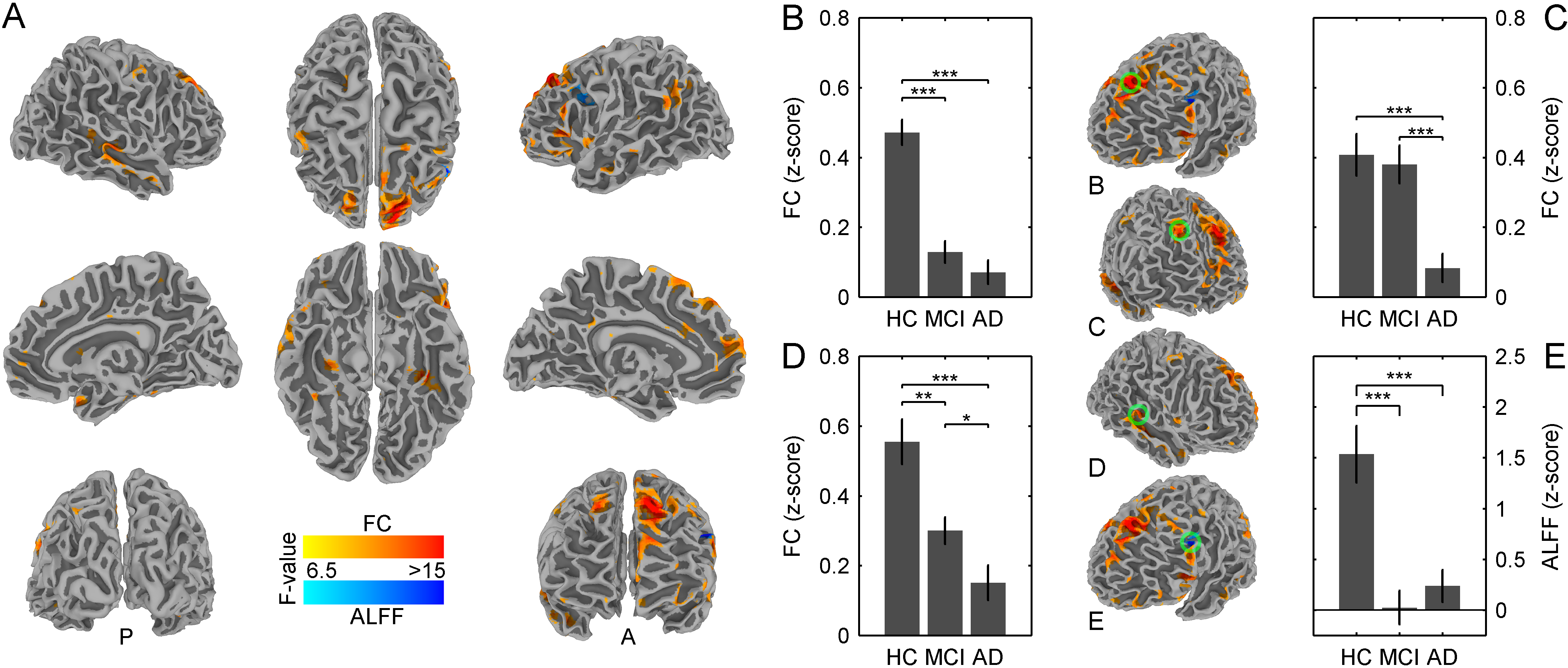

Supplement: S2 Fig — (A) f-statistic maps showing significant detectable changes of FC (hot colors) and ALFF values (cold colors) among the three groups of subjects (HC, MCI and AD). Of note, only one lateralized frontal region showed a significant change in ALFF among groups. Results were obtained via one-way ANOVA, separately accomplished for FC and ALFF data. The statistical threshold was set at f > 6.48 (p < 0.05, corrected). (B-E) Post-hoc analyses of the most relevant clusters belonging to FC (B-D) and ALFF (E) changes. For each cluster, group comparisons were performed via two-sample, two-tailed t-tests on the voxel showing the local maximum of f-values. *, p < 0.05; **, p < 0.01; ***, p < 0.001; A, anterior; P, posterior. (TIF) [file pone.0120988.s002.tif]

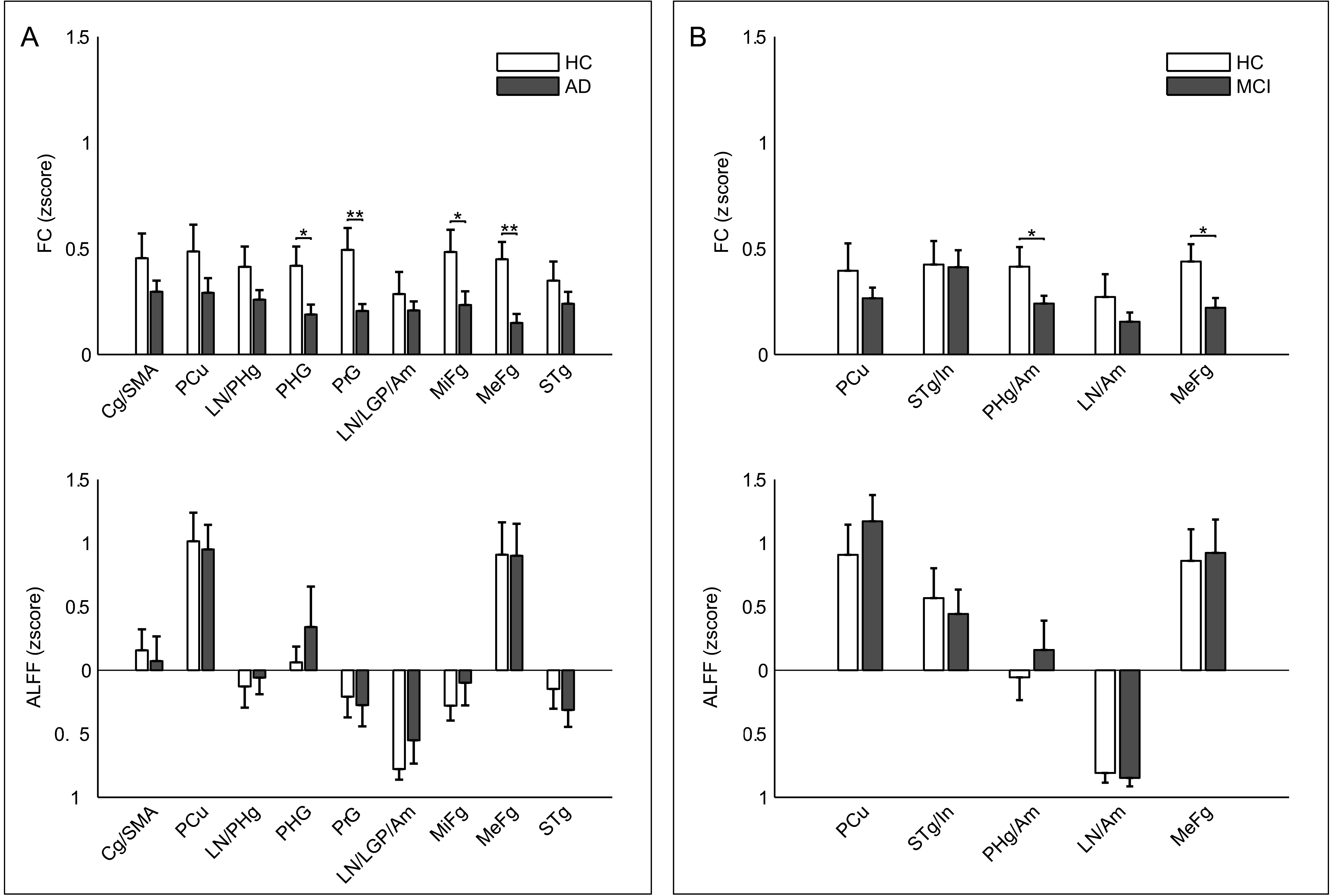

Supplement: S3 Fig — FC and ALFF values were averaged in the significant clusters resulting from t-contrasts HC vs AD (A) and HC vs MCI (B) both computed in the FC vs ALFF full-band model (see Fig 4). For the details of the regions, see Tables 2 and 3. *, p < 0.05; **, p < 0.01 (two-sample, one-tailed t-test); Cg, cingulate gyrus; SMA, supplementary motor area; PCu, precuneus; LN, lentiform nucleus; PHg, parahippocampal gyrus; PrG, precentral gyrus; LGP, lateral globus pallidus; Am, amygdala; MiFg, middle frontal gyrus; MeFg, medial frontal gyrus; STg, superior temporal gyrus; In, Insula. (TIF) [file pone.0120988.s003.tif]
